# Genome Sequence of an *Okra Leaf Curl Virus* from Egypt

**DOI:** 10.1128/MRA.00533-21

**Published:** 2021-07-22

**Authors:** Mahmoud Magdy, Andrew S. Appiah, Samah M. Rizk, Hagar T. Elhifnawi, Benjamin Karikari, John K. Ahiakpa, Noha K. El-Dougdoug

**Affiliations:** a Genetics Department, Faculty of Agriculture, Ain Shams University, Cairo, Egypt; b Biotechnology and Nuclear Agriculture Research Institute, Ghana Atomic Energy Commission, Accra, Ghana; c Department of Crop Science, University for Development Studies, Tamale, Ghana; d Research Desk Consulting Ltd., Kwabenya-Accra, Ghana; e Botany and Microbiology Department, Faculty of Science, Benha University, Benha, Egypt; KU Leuven

## Abstract

A complete *Okra leaf curl virus* DNA-A was sequenced from okra in Egypt. Here, we report the complete genome sequence of this monopartite virus, comprising 2,764 bp and encoding 6 open reading frames (ORFs) with a GC content of 44.6% and 88.3% similarity to a virus reported earlier from Cameroon.

## ANNOUNCEMENT

Okra (Abelmoschus esculentus L. Moench) is cultivated for its pods as a food source throughout Egypt, which is currently ranked the ninth country in world okra production (1). Several *Begomovirus* species (family *Geminiviridae*) were identified in various Malvaceae plants (2–4). Fauquet et al. (5) reported the susceptibility of okra to eight *Begomovirus* species, and among them are *Okra leaf curl virus* (OLCV; monopartite), *Okra yellow vein mosaic virus* (OYVMV; monopartite), and *Okra enation leaf curl virus* (OELCuV; monopartite), which contribute between 30% and 100% yield losses in okra (4, 6), particularly under field conditions (7, 8). The OLCV is transmitted by the whitefly (Bemisia tabaci) and causes the okra leaf curl disease characterized by leaf curling, crumpling, and malformation, severe stunting; vein enation, and necrosis (6). Members of the genus *Begomovirus* have circular single-stranded DNA (ssDNA) genomes which are either monopartite (one ∼2.8-kb DNA component) or bipartite (two ∼2.6-kb DNA components). They are the largest genus of the family *Geminiviridae* and cause economically significant diseases of many vegetable and fiber crops (9, 10). In this study, we assembled and reported a new complete OLCV DNA-A genome sequence identified in okra cultivated in the open experimental field of Ain Shams University, Cairo, Egypt.

DNA extraction was performed using a DNeasy plant minikit (Qiagen, USA) on 0.6 g infected okra leaf. An Illumina paired-end DNA library was constructed using the TruSeq preparation kit (Illumina) with an average insert size of 350 bp and sequenced using the Illumina HiSeq 4000 platform (Novogene, China) to generate paired-end 150-bp reads. The single-contig assembly approach (11, 12) was employed, as a 5% portion of the total reads were *de novo* assembled. All the circular contigs were subjected to a search at the online NCBI database (nucleotide collection [nonredundant nucleotide] using Megablast set to okra) to identify viral-related circular sequences. The clean pair-end reads were remapped to the single viral contig with 15 iterations (times), rechecked for variants, and annotated based on the BLAST results. The read trimming (trim-end tool was set at 0.05 error probability limit), *de novo* assembly, mapping, and annotation were performed in Geneious prime using the default parameters unless otherwise specified (13).

**FIG 1:**
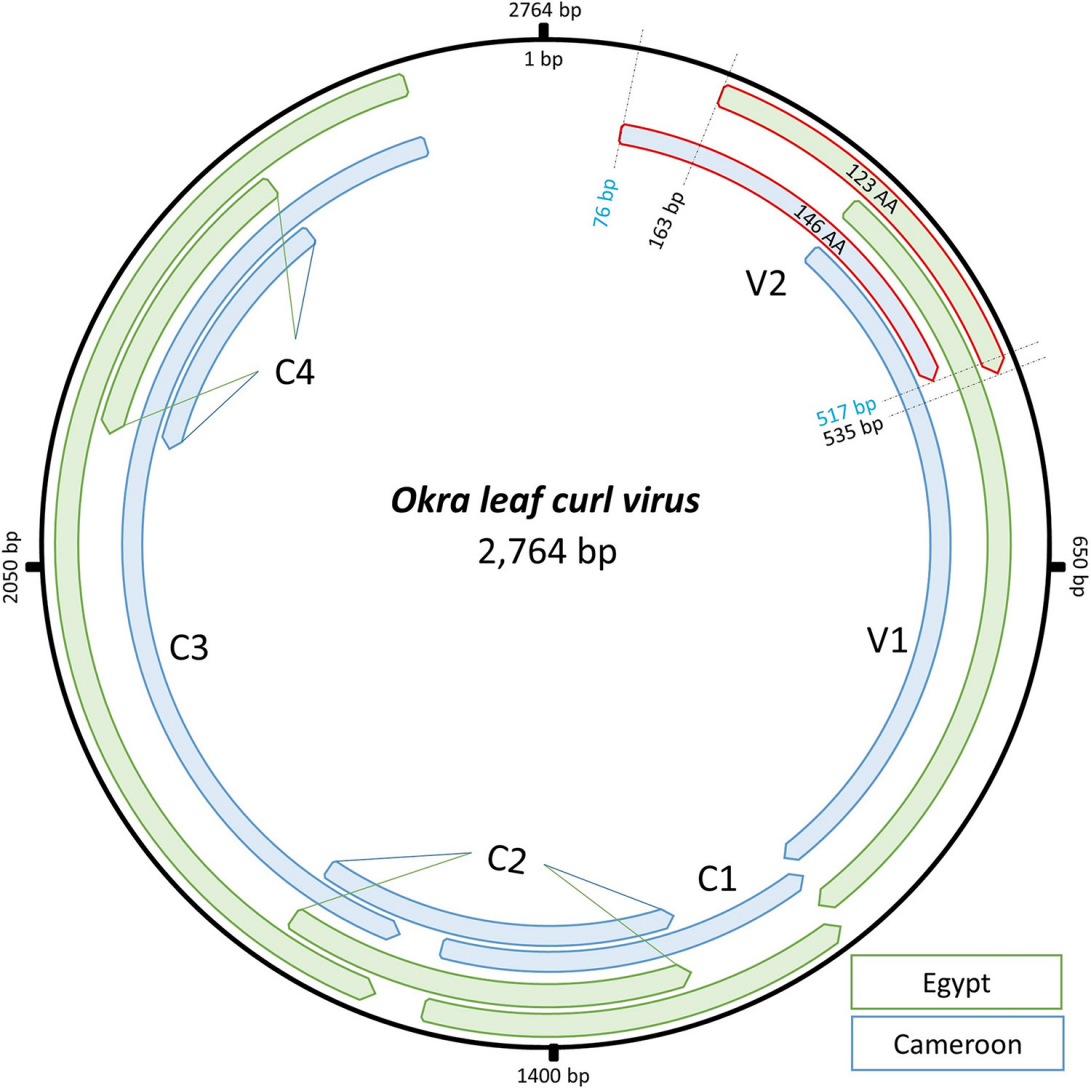
Comparative circular genetic map of *Okra leaf curl virus*. The OLCV genome reported from Egypt (green tags) versus the one reported from Cameroon (blue tags). Six annotated ORFs are shown; for the ORF “V2,” the differences in the annotation intervals between both genomes are detailed in numbers.

Out of 86 million clean paired-end reads, 83 circular contigs were assembled. A single circular contig was highly similar (88.3%; query coverage, 100%) to the OLCV reported from Cameroon (GenBank accession number NC_014745; GC content of 43.5%). The genome was 2,764 bp in length, with 6 open reading frames (ORFs). Four reversely oriented ORFs, namely, the replication association protein (C1; 1,089 bp; 363 amino acids [aa]), transcriptional activator protein (C2; 405 bp; 135 aa), replication enhancement protein (C3; 402 bp; 134 aa), and a hypothetical protein (C4; 294 bp; 98 aa), and two forwardly oriented ORFs, specifically, coat protein (V1; 777 bp; 259 aa) and precoated protein (V2; 369 bp; 123 aa), were annotated (Fig. 1). Compared with the OLCV reported from Cameroon, the Egyptian strain was distinguished by 216 point mutations. The major portion of variants were found in the coat protein gene V1 (128 variant sites; 22.3% dissimilarity) and the precoat protein V2 (63 variant sites; 28% dissimilarity), with 35 shared variants in the overlapping region between V1 and V2, while C genes showed ≤5% of variation. Specifically, three point mutations in the V2 gene (78 G > T, 79 C > A, and 132 T > A) led to a premature stop codon and thus a reduction in the precoated protein size from 146 to 123 aa (Fig. 1). These data may serve as a useful genomic resource for future comparative genomics and phylogeography analysis on OLCV.

### Data availability.

The OLCV genome isolated from Egypt (O12) has been deposited in the NCBI GenBank database under the accession number MW015091, and the raw fastq reads were deposited in the NCBI SRA database under the accession number SRR12963442.
